# Newly qualified nurses’ experiences of transition to professional practice in the context of COVID-19: a qualitative study

**DOI:** 10.1177/17449871251380463

**Published:** 2025-11-25

**Authors:** Catharine Jenkins, Vanda Carter, Analisa Smythe

**Affiliations:** Associate Professor, Department of MH and LD nursing, Birmingham City University, Birmingham, UK; Nurse Researcher, School of Health and Social Care, University of Staffordshire, Staffordshire, UK; Visiting Fellow, School of Health and Social Care, University of Staffordshire, Staffordshire, UK

**Keywords:** management and leadership, nurses, practice development, qualitative, workforce and employment

## Abstract

**Background:**

The transition from student nurse to newly registered professional can be challenging, sometimes leading newly qualified nurses to reconsider their career choice. Workforce shortages and the experience and aftermath of the COVID-19 pandemic may have exacerbated the pressures on recent cohorts.

**Aim:**

To gain understanding of experiences of transition into professional practice in an English NHS Trust during the COVID-19 pandemic.

**Methods:**

The objectives of this study were met using a qualitative, exploratory, intrinsic case study design. A convenience sample of 16 newly qualified nurses were recruited. Transcripts were analysed as individual interviews then subjected to across group analysis. Data analysis was conducted independently by the research team members. Data were subjected to qualitative content analysis.

**Results:**

The findings describe the nurses’ experiences of transition to professional practice during the pandemic, there were two main themes: the transition experience and growing into the role.

**Conclusions:**

The study offers insights into experiences of transition in the context of the COVID-19 pandemic. The study demonstrates that workplace culture has a significant impact on transition experiences. Emotionally safe, supportive environments are a prerequisite for an effective transition, allowing development of the confidence and skills necessary for independent practice and a successful career.

## Introduction

The transition from nursing student to newly qualified nurse (NQN) can be challenging (Smythe and Carter, 2022). In the United Kingdon (UK) preceptorship is a structured programme which offers support and guidance for all newly registered practitioners to build confidence and competence during the first-year post-registration (NHS England, 2022). Despite the implementation of formal transition programmes, NQNs’ experience has been described as comprising of overlapping stages; Kramer (1974) identified ‘honeymoon, shock, recovery and resolution’, Duchscher (2012) ‘Doing, Being and Knowing’ as stages of ‘Becoming’, whereas Hsiao et al. (2021) identified ‘hesitation, psychological preparation, development and appreciation’. Duchscher (2012) also identified ‘Transition shock’, a demanding and emotional experience resulting from the challenges of adjusting from the relatively protected and supported position of student nurse to the highly responsible, complex role of a registered nurse (Table 1).

**Table 1.** Stages of transition: Three models.

Kramer (1974)	Duchscher (2012)	Hsiao et al. (2021)
Honeymoon	Doing	Hesitation
Shock	Being	Psychological preparation
Recovery	Knowing	Development
Resolution	Becoming	Appreciation

Undergraduate education is designed to prepare nurses; however, even before the pandemic, many reported feeling ill-equipped for the role, leading to stress, job dissatisfaction and consequent intention to leave the profession (Collard et al., 2020). Examples of contributing factors included limited clinical training (Wilkinson and Hayward, 2017), NQNs facing unrealistic expectations from senior staff (Regan et al., 2017), and feeling under-supported (Collard et al., 2020).

There is a global nursing shortage with an ongoing projected shortfall (World Health Organization, 2020). After qualification, NQNs in the UK are usually supported through mentorship and a structured period of preceptorship (Health Education England (HEE), 2020). Low staffing levels impact on quality of care and increase pressures for staff (Care Quality Commission, 2019); supporting an NQN adds another element of demand (Smythe and Carter, 2022). There are many perceived benefits attributed to preceptorship programmes, once well-embedded in organisational culture (Crawford et al, 2000; NHS Employers, 2020). These include successful recruitment and retention of staff and how it positively contributes towards the well-being and resilience of the workforce (O’Driscoll et al., 2022) and NQNs recognise the positive impact of this support (Wilkinson and Hayward, 2017). However, the nature of nursing practice during the pandemic added huge additional pressures (Hernández-Bojorge et al., 2024). Much of the existing research into the transition experiences of NQNs has been conducted internationally (Hawkins et al., 2018), and pre-pandemic. For example, Hung et al. (2018), in China, noted that NQNs were stressed by ‘reality shock’, related to the complex needs of patients and the heavy workload, sometimes exacerbated by undermining attitudes and behaviour from their more experienced colleagues. However, most of these NQNs were well supported through a mentorship programme, so adapted and gradually grew in confidence. In the Netherlands, ten Hoeve et al. (2018) similarly found the transition stressful, but positive experiences and relationships with peers and senior colleagues were key to successful adaptation and development of commitment to the profession.

Research investigating the impact of the pandemic on newly qualified nurses is still emerging. Registered nurses worldwide have been traumatised by pandemic-related factors including the high level of patients’ suffering, lower quality care, redeployment and uncertainty, moral distress, feeling overwhelmed by the workload and fear for their own and family members’ safety (Gedik et al., 2023;Hernández-Bojorge et al., 2024). This was particularly challenging for younger, less experienced nurses (Hernández-Bojorge et al., 2024). Pagnotta et al. (2023), in Canada, identified that student nurses approaching registration experienced high levels of stress because of uncertainty, inconsistent learning opportunities, moral dilemmas and threat to their own safety in the workplace. NQNs in Sweden (Carnesten et al., 2022) reported working beyond their capabilities and being overwhelmed by feelings of being out of control, unprepared, distressed by inadequate standards of care and exhausted, whereas in China, Wang et al. (2023) reported high levels of ‘transition shock’ and Zeng et al. (2023) found compassion fatigue, burnout, and secondary traumatic stress.

The current study was conducted in an NHS Trust in England. The COVID-19 pandemic significantly impacted on NQN training, compromising preparation with reduced clinical placements, shifts towards remote learning and reduced access to preceptorship, amplifying existing issues (Duchscher et al. 2021; Merino-Godoy et al., 2024). Students who graduated during and post-pandemic reported concerns about the lack of Higher Education Institution support, anxiety about catching up on their academic studies and concerns about clinical abilities and skillsets (Health Education England, 2020).

A recent literature review suggested that workplace culture had a significant impact on NQNs’ experiences; supportive socialisation in an emotionally safe environment was found to aid development of competence and confidence (Smythe and Carter, 2022). This study builds on such previous evidence by exploring the experiences of NQNs in England during and immediately post-pandemic, specifically examining the additional impact of COVID-19 pressures on the transition experience, including how the dynamics of interpersonal relationships within a workplace culture might affect NQNs. Learning from these experiences is important for minimising ongoing consequences for those affected and may be relevant in cases of any future health emergencies (Djukic et al., 2025). This research could inform policy and practice around complementary educational and workplace interventions designed to facilitate successful transition to registered practice in the context of global healthcare recovery from pandemic-related stressors.

## Methodology

### Aims and objectives

The study aimed to gain understanding of NQNs’ experiences of transition into professional practice. The study was planned pre-pandemic; however, it affected participants’ experiences, so it was important to include its impact on the transition experience. The objectives were to identify NQNs expectations of transition and to determine the barriers and facilitators to successful transition, including the contributions of workplace induction and preceptorship support.

## Methods

The aim and objectives of this study were met using a qualitative, exploratory, intrinsic case study design, underpinned by a constructivist philosophical positioning (Harrison et al., 2017). Case studies using qualitative methods are valuable when the questions posed require in-depth investigation of an issue in its natural, real-life context (Alorrie 2004). An intrinsic case study is a type of case study research where the researcher focuses on a particular case because it is inherently interesting or important (Stake, 1995). The intrinsic case study design was valuable as it captured the complexity of the transition experience during a rare event, the COVID-19 pandemic. The overall aim of this study is to gain insight into and understanding of NQNs’ experiences of transition to professional practice. The ‘case’ in this study centres on NQNs’ experiences of transitioning to professional practice during the pandemic. Recruitment took place within an integrated NHS Trust. The Consolidated Criteria for Reporting Qualitative Research (COREQ) were followed (Tong et al., 2007).

### Setting and recruitment

A purposive sample of 16 NQNs were recruited from a broad range of settings across the NHS Trust, including the hospital and community (Campbell et al 2020). Data saturation and information power were considered in the study design. Recruitment during COVID-19 restrictions was problematic. Flexibility and safety were key considerations. Data saturation and information power were reached organically in the context of unprecedented circumstances (Braun and Clarke, 2022; Malterud et al., 2021). The researchers felt that sufficient depth of information was attained to meet the study aims and objectives.

Criteria for participation were graduation from a UK university and employment as a registered nurse for a maximum of 1 year. Participants were excluded if they were undergoing any form of disciplinary proceedings. The study was publicised via the Trust communications department and internal educational meetings. NQNs who wished to join the study were asked to email the Chief Investigator. Subsequently, they received an email containing a letter of invitation and a participant information sheet explaining the study. Participation involved engagement in a face-to-face or remote individual or group interview (Table 2).

**Table 2.** Mode of interview.

Interview number	Mode of interview	Number of participants
Interview 1	Face to face	1
Interview 2	MS Teams	2
Interview 3	MS Teams	3
Interview 4	MS Teams	3
Interview 5	Face to Face	2
Interview 6	Face to Face	2
Interview 7	MS Teams	2
Interview 8	MS Teams	1

### Data collection

An interview topic guide was used to ensure fulfilment of the study aims (Table 3). The interview schedule was not piloted; and is acknowledged as a potential limitation. Interviews lasted between 45 and 120 minutes and were audio-recorded. Field notes were made following each interview. Data were collected between November 2021 and June 2022 by one female nurse researcher, educated to PhD level, who was unfamiliar to the participants. COVID-19 pandemic pressures posed significant challenges to recruitment. Adaptations had to be made to accommodate the imposed restrictions, for example, changes to the mode of interview. Group interviews were replaced by individual interviews due to the recruitment challenges experienced. The different modes of interview may have influenced the participants’ responses. Being part of a group may have enhanced conversations and recall about an experience, equally one to one interviews may remove any inhibitions of having others present.

**Table 3.** Focused interview topic guide.

Introductory questions (get participants to start and think about their connection with the topic)
• When did you qualify as a registered nurse?
• For how long have you worked at your place of work?
• What area of practice (specialty) do you work in and why?
Transition questions (serve as a link between the introductory questions and the key questions)
• In what ways do you feel your pre-registration training prepared you for your role as a registered nurse?
• Did you have any expectations of what transition from student nurse to registered nurse might be like?
• The final pre-registration practice placement has been described as ‘a dress rehearsal for the real world of work.’ What are your thoughts about this?
Key questions (questions that drive the study)
• Can you tell us a little bit about your induction to the Trust as a new member of staff/newly qualified nurse?
• How would you describe your first months/year as a newly qualified nurse?
• Based on your experience, what was the provision of support like?
• Did you receive preceptorship? If yes, how did this work?
• What would you say has helped your transition?
• Has anything negatively affected your transition?
• What (if anything) might have improved your transition experience?
• Have your work place/career plans changed in any way since becoming a registered nurse?
Why? In what ways?
Ending questions (bring closure to the discussion)
• We were interested in finding out about your experiences of transition from student to registered nurse. Is there anything that you came along wanting to say that you haven’t had a chance to say?

### Data analysis

Interviews were transcribed verbatim and quality checked by two members of the research team. Data were subjected to inductive qualitative content analysis (Hsieh and Shannon, 2005) This involved three stages: reading and re-reading the interviews to get a sense of the data as whole, labelling condensed meaning units by formulating codes, and grouping these codes into categories and themes. Data analysis was carried out independently by all three members of the research team. The researchers met at regular intervals to reflect on analysis and themes and reach consensus.

### Trustworthiness

Several techniques were used to ensure trustworthiness of the study findings (Lincoln and Guba, 1985). Credibility was addressed through application of quotations to support interpretations. A single researcher carried out all the interviews, which offered consistency in data collection. The researcher held a senior role in an National Health Service (NHS) organisation and was known to some of the participants. This may have influenced the participants’ narrative and the information they shared. Dependability was assured by providing a detailed account of the research process, together with record-keeping and internal reporting, serving as an audit trail. The researchers engaged in ongoing reflection throughout the research process to ensure confirmability and maintain reflexivity (Arber et al 2006). A third researcher, external to the organisation, joined the study prior to data analysis, to promote impartiality. Transferability was enhanced through providing a rich description of the findings with reference to the context and by providing a clear outline of the study methods. Participants were aware the study was unfunded; the organisations involved wished to improve the NQN transition experience and subsequently retention.

### Ethical considerations

Approval was obtained from the Health Research Authority (HRA; IRAS ID: 295134). Informed consent was received from participants prior to all interviews. Data were stored securely and anonymised during transcription. To protect anonymity, interviews were carried out away from the ward area. In case participants required additional support, they were made aware of the Trusts’ workplace support, which offers free 24-hour advice and counselling.

## Results

All the participants apart from one were female, ages ranged from mid-20s to mid-30s. Participants were from a mix of ethnicities. They had all been qualified for between 3 and 12 months.

The analysis was informed by a literature review, which was carried prior to the study (Smythe and Carter, 2022). The review was used as the basis for the coding framework; this was modified as new themes emerged. The coding framework included: preparation for real-world practice, the transition experience, the role, and preceptorship and support. The results describe the nurses’ experiences of transition to professional practice, during the COVID-19 pandemic and soon afterwards. Two themes were identified: The transition experience (theme 1) which included five subthemes: the pandemic, feeling overwhelmed, interaction with colleagues, organisational stress and supportive factors. As in pre-pandemic studies, the transition experience was found to be immensely stressful, but more so due to the additional stressors, which impeded both preparation and support. ‘Growing into the role’ (Theme 2) included two subthemes, learning opportunities and increased confidence. The NQNs mostly, described this process as having more significant obstacles, and was more complex, than reported pre-pandemic. The subthemes highlight specific aspects of transition and growth into the role (Table 4).

**Table 4.** Main themes and subthemes.

Main themes	Subthemes
The transition experience	The pandemic Feeling overwhelmed Interactions with colleagues Organisational stressors Supportive factors
Growing into the role	Learning opportunities Increased confidence

### The transition experience

#### The pandemic

There was no sense of a ‘honeymoon period’ or psychological preparation for this group of NQNs, who spoke about feeling unprepared, with the pandemic impacting on transition experiences. Their student university cohorts appeared to have been impacted by limited placement and learning opportunities, so even before transition into registered practice, they were at an experiential disadvantage:

*‘I think COVID really affected our studies, our last year’s studies – because we had nearly all online classes, and then it was so hard. We couldn’t even meet our friends. When we were at the University we can’t go to the library, we could still see it online but then I find reading books and taking notes from books . . . and also I just like face to face teaching rather than online .’*– Interview 1 P2.

The pandemic also appeared to exacerbate workload pressures:

*‘At times like especially during this COVID period it’s been like din din din . . . one after the other, one after the other.’* – Interview 2, P1.

These quotes explicitly illustrate perceptions of COVID-19 as the context of reduced confidence and higher workload. This context was all pervasive and the demands the NQNs faced, and their emotional responses, reflected this, whether directly acknowledged, or not.

### Feeling overwhelmed

The first stage of transition has also been described as ‘reality shock’. The shock was particularly challenging for this cohort, who conveyed a strong sense of hesitation as they stepped into practice. For most participants, the first months were very stressful:

*‘We have the base knowledge but that bears no resemblance to what you are expected to do when you actually start . . . to start with it is like a rabbit in headlights.’* – Interview 6 P3.

However, the demands on staff were like none seen before. It was common for the NQNs, especially those who were parents, to find extended shift patterns impacted negatively on their family lives:

*‘I am lucky because my mum supports me with the kids, but my five o’clock shift then turns into an eight o’clock finish.’* – Interview 5 P2.

The nature of patients’ needs and expectations, and the lack of staff, resulted in immense pressure of work and a pattern of poor self-care for staff. For example, being unable to go to the toilet, or take any meal breaks:

*‘I need to eat my breakfast before I start, otherwise I am not going to eat until 3 o’clock, I am going to collapse. . . hoping for a second break, you never have a second break.’* – Interview 7 P3.

The NQNs described the toll of expending their energy at work, to the extent that there is none left for themselves and their family when they get home:

*‘I get home, and I have got no compassion left for anybody, because I have listened to people so much all day, I am like really, I am not interested. I will talk to my other half but then anybody else I don’t really want to talk to. Sometimes I feel just talked out, talking to patients . . . My daughter says, ‘I could be dead in this house’ (laughs).’* – Interview 7 P2.

Negative experiences left several NQNs feeling overwhelmed, concerned for their own physical and emotional well-being. Sleep was described as a particular issue for many as they processed the difficult events experienced on shift:

*‘That night I couldn’t sleep; I took it home. The next day I called in sick in the morning to reflect on the whole night. But then what I keep asking myself is that how had it been I didn’t recognise those signs, where would I be and also the patient. Where would the patient be? If he had neurological damage, what kind of life that person would have?.’* – Interview 2 P3.

For some, such experiences affected their mental well-being, even to the extent of not being able to return to work:

*‘I was so upset after that shift; I couldn’t go back. I could not go back, and I haven’t been back. I went off sick. My anxiety was through the roof. . .’* – Interview 3, P2

These examples indicate the emotional consequences of the NQNs’ experiences. The usual means of addressing challenges to well-being, such as restorative sleep, eating well, taking breaks, talking with loved ones, seemed unavailable and beyond the scope of self-help techniques due to the nature of patients’ extremely complex needs.

#### Interactions with colleagues

Support from colleagues is key in developing skills and confidence for a new role and can aid recovery from the initial reality shock. The NQNs reported the time of transition in very emotional language, reflecting the intensity of their experiences. Interactions with colleagues and support within the workplace appeared to have a significant impact on their experience:

*‘I would hate to feel how a newly qualified would be if the team wasn’t so approachable . . . I do think I have the best team in the Trust because they are so accommodating, everybody celebrates all the little wins of everybody, and if somebody is feeling really down, we are dealing with that as a team, we will offer advice and pick you up . . ..’* – Interview 5 P1.

However, it was suggested that providing support came at a cost to the more experienced nurses, who had workloads that they needed to manage:

*‘They have a bay of their own patients, so they are not as available as they used to be, that is the only thing. They are still supportive; it is just that you can tell you are adding extra stress to them when you do need something.’* – Interview 5 P3.

Interactions were taking place in the context of very high stress levels, due to caring for extremely ill patients. Threat to registration and perceptions of individual accountability appeared to be associated with self-protective behaviour, both by more experienced staff and NQNs:

*‘Probably their own patient is triggering sepsis, so will they tell you, no. I know yours is sepsis too but mine is sepsis too, so she has to cover her back because she’s got her PIN [registration]to protect.’* – P2 Interview 2.

The NQNs often empathised with their more experienced nursing colleagues; however unfortunately, for some NQNs, colleagues’ behaviour was unsupportive, undermining and even hurtful towards them. In the extract below, a participant discusses colleagues’ behaviour towards each other:

*‘You expect nurses to be some of the most compassionate, kind people – but especially critical care nurses, and they are just brutal, brutal.’* – Interview 3 P2.

Disrespectful or undermining communication from colleagues was often described as harmful:

*‘She just shouted, why did . . .? I couldn’t do anything. The way we were shouted at, everyone was listening, the cleaners, the HCA’s [Health Care Assistants], the nurses who had come for their day shift, the ward clerks were all on there and aww. . .’* – Interview 4 P4.

When NQN’s felt unsupported or requests for help were denied, this made nurses feel incompetent and question their own abilities and then find it difficult to delegate duties to colleagues themselves. This appeared to create potential conflict between Health Care Assistants (HCAs) (unregistered health care staff that support nurses with basic delegated patient care) and the NQNs:

*‘I told them that I still had work to do, they feel that we should be washing patients and still having to do our work. It is a lot, because they go and just sit down, and they are on their phones, and we are still doing our work. I find that is where a lot of nurses, they don’t want to deal with it.’*– Interview 4, P3.

The NQNs recognised the influence of relationships with experienced colleagues on their own confidence, competence, and emotional well-being. Patients’ needs were complex and demanded high levels of attention, so they could understand the reasons why their colleagues could not offer the level of support they required, but this did not make it easier.

### Organisational stressors

Workload stressors were compounded by managerial, organisational, and administrative challenges. Staff shortages combined with the extensive needs of patients caused the NQNs considerable concern:

*‘They are sending us newly qualified to wards whereby the staffing levels are very low. The staff are very nice but they themselves are struggling. So don’t expect staff to come and look after you, because you are newly qualified. In fact, they think you’re coming to rescue them.’* – Interview 2 P3.

The NQNs noted that the level of pay was not sufficient compensation for the demands placed on them by their employers; furthermore, it did not reflect their effort and responsibilities:

*‘Not that you should be money led but when you come in and you are so tired you can’t even do anything else, and then by the end of the month as soon as it comes in, it goes out. By the middle of the month, you have got nothing.’* – Interview 6 P2.

Low pay and stressors led many to consider alternative means of earning a living, for example:

*‘Without this stress I can go on and do a different job anyway. And get a much better decent salary than what I’m getting now. And after studying for 3 years. And all the pressures that comes with the job.’* – Interview 2, P2

High workload in combination with perceived poor pay led to feelings of exploitation and anger. The NQNs expressed how they should feel cared-for, but instead they often seemed to feel that their work and their experiences were unrecognised and unacknowledged.

### Supportive factors

A supportive team and good leadership were identified as making the transition easier. In addition to the interpersonal, there were other workplace factors such as suitable staffing levels and setting:

*‘Okay, we get busy, but it is managed, it is managed quite well, we only have the amount of people that we can actually cope with.*’ Interview 6 P3.

NQNs’ preparation focusing on transition and practice placement experience were perceived as helpful. Experiences of being supernumerary also helped, depending on the time given, which ranged from 3 to 6 weeks:

*‘And because I knew I was supernumerary I knew I had that bit of support, if that makes sense. It wasn’t for very long, but it helped.’* Interview 5 P3.

The NQNs’ experiences of positive and negative elements of transition gave them insight into potential changes, interventions and support that could make a difference for others. Many mentioned flexible shift patterns and longer supernumerary status arrangements.

These issues led many participants to reflect on how they could reduce stress and improve their well-being. The NQNs’ words reveal their insight into and empathy for their colleagues’ positions, given the context and overwhelming impact of the pandemic. While much of their experience emphasises emotional struggles, they were also able to share more optimistic reflections and recognise helpful factors. The ‘Transition’ theme covered an extensive amount of content, bravely shared by the NQNs. Although it is saddening at times to read their words, they also gave suggestions for how things could change so that future NQNs’ experiences might be different.

### Growing into the role

#### Learning opportunities

This stage of transition might be considered a process of recovery. Participants valued the networking and the learning opportunities provided by a well-organised preceptorship programme, which helped them adapt:

*‘You know, it is like lots of new things you learn. You do learn leadership in the University, but then it is not that you will take from here, I think it is a bit more that you can take up from your work. It’s definitely helpful.’* Interview 1 P2.

However, barriers to accessing preceptorship were seen to include poor organisation of the programme and ‘backfill’ arrangements, staff shortages, limited access to preceptors, as well as confusion about the educator roles:

*‘I haven’t done the Preceptorship, . . . we did have the emails for that but then obviously like she said, they are short staffed so you can’t really get there. . .’* Interview 4 P2.

Participants were often given a name of an allocated preceptor in their ward when joining a team, but contact was often minimal:

*‘She never came and approached me so the next time I saw her I said ‘are we supposed to do something together’, she said ‘oh don’t worry its fine, you don’t need to worry about that’. Nothing happened.’* Interview 7 P3.

Participants often struggled to book a place on the preceptorship programme, finding time to attend was also an issue for many. Some participants suggested a lack of commitment and capacity to undertake the role of preceptor:

*‘I think it does depend on which nurse you are assigned to work with, because some are brilliant, and some are – it is not their thing teaching. I think with all the NMC [Nursing and Midwifery Council] things now they are forced into that role. Some people just can’t teach, it’s just not in them. They can be a very good nurse but as for teaching . . .’* Interview 6 P3.

Others explained that their preceptorship opportunity came too late, after 6 months in the role, so the programme was no longer relevant :

*‘I did my Preceptor study days recently because that time there was no-one. . .. So, I’m just thinking whether I have to do them all, because it’s like making the transition is one, and then learning to learn, so I am thinking whether I have to do this or not, because I’m already one year in.’* Interview 1, P1.

It seemed that engagement with preceptorship faced barriers from both parties, exacerbated, in the context of the pandemic, by staff shortages.

#### Increased confidence

Despite this context and the immense challenges faced by all staff, positive experiences could have an empowering effect, as recovery moved towards a sense of resolution and appreciation:

*‘Some of the staff were really helpful, they just helped me so much to take my transition from student to qualified nurse. So, I have learned lots of new skills from there.’* Interview 1 P2.

Confidence was also strengthened through professional development; this was in the form of personal study and learning on the job:

*‘Aany medication I would write that down and I would put a picture next to it and I would put little quotes next to it so I know exactly what to do the next time it comes. . ... But I had to literally go away and learn it myself, educate myself.’* Interview 3 P4.

For some a poor relationship rooted in disrespectful communication could lead to a hesitant approach. Lack of confidence was then associated with reluctance to speak up or unwillingness to escalate untoward incidents:

*‘I didn’t have the confidence escalating that situation, and I thought well she is a Senior Outreach person, she knows what is supposed to be followed.’* Interview 8 P2.

As the NQNs’ confidence grew, some expressed a wish to continue working in their current areas of practice and were unwilling to move area until they felt they had mastered their current role:

*‘As your confidence builds up you begin to kind of enjoy the role. . . I kept pushing until I could feel my confidence had built up I don’t know everything about my workplace but I know what I am doing. So, with that, yes I can honestly say I am enjoying (the role now). . ..’* Interview 4, P4.

These examples indicate that despite the stressors of the early months, participants appeared to gradually adjust to their new roles and expressed feeling positive about their professional growth.

## Discussion

The challenging nature of the transition into practice, or ‘transition shock’ (Duchscher and Windey, 2018) that these findings convey echo previous studies (Kukkonen et al., 2025; Labrague and de los Santos, 2020, Lee and Gagne, 2022, Sampson et al., 2020). For this group of NQNs, the ongoing pandemic-related workload pressures in combination with staffing problems led to an overwhelmingly exhausting transition, unlike any experienced by previous generations, whose experiences might have fitted more neatly into theoretical stages (Duchscher, 2012; Hsiao et al., 2021; Kramer, 1974). For example, our findings indicated none of the ‘honeymoon’ stage that Kramer (1974) identified. The NQN participants’ experiences of ‘Doing’ were perhaps even more intimidating and overwhelming than Duchscher (2012) described, given the pandemic-related exacerbation of unpredictability in clinical practice. Similarly, their reports of doubting and questioning their own and others’ abilities, and then growing in confidence, may reflect something of Duchscher’s (2012) ‘Being’ stage. However, this appeared to be delayed for our participants when compared to the anticipated timescale of around 5 months post-registration.

Development of confidence and job satisfaction is dependent on relationships with senior staff (ten Hoeve et al., 2018), but for this cohort of NQNs, senior staff were experiencing huge pressures themselves, so additional demands for emotional support from junior colleagues were perhaps perceived as having life or death consequences, should they leave their seriously ill patient. Dewey et al. (2020) suggested that clinicians’ feelings around lack of control were associated with burnout. Senior staff were therefore probably psychologically incapable of providing emotional and practical guidance for the NQNs in such demanding circumstances.

The period immediately post-qualification had already been recognised as leading to significant emotional distress (Sampson et al., 2020); the most difficult time for a nurse is within the first 12-months post-qualification (Krut et al., 2021). In this study, many NQN participants identified that support and guidance was available in theory, but due to the nature of unit pressures during and immediately following the pandemic, they were unable to access such provision. Nevertheless, given the level of stress described by participants, it is clear that high levels of resilience were required to negotiate every day professional practice. This was extremely challenging and at times they appeared to feel that they were not coping, but strategies including indirectly or covertly seeking guidance (e.g. by initiating a conversation as an excuse to generate advice), family support, and even just the passage of time and growth of confidence aided them in navigating this extremely difficult episode.

The participants in the current study had a unique experience in transitioning into practice during the pandemic, suggested as being associated with higher levels of trauma for nursing staff (Aukerman et al., 2022). In a recent literature review, Mammen et al. (2023) (all data collected pre-COVID) found rude, disrespectful, bullying and undermining forms of communication were harmful and widespread among nurses. A recent case study by NHS England (2023) noted that development opportunities for NQNs were severely impacted by the pandemic as more experienced nurses were redeployed to intensive care, leaving fewer, less experienced colleagues to support NQNs. Recent post-pandemic studies not only identified increased vulnerability in NQNs (Aukerman et al., 2022; Brook et al., 2023) but also perceived subsequent resilience (Godbold et al., 2022), which is reflected in this study. Developing resilience to challenge should not be down to the individual nurse but be engendered by a supportive environment.

Difficult transitions may be regarded as a ‘rite of passage’, or tradition that every nurse experiences (Darbyshire et al., 2019). This assumption is supported by evidence; a review found the majority of NQNs experienced bullying at work (Alshawush et al., 2021). However, this ignores the context of this post-COVID-19 generation, while avoiding a reckoning with the nurses ‘eat their young’ dynamic, which should be unacceptable professional behaviour at any time (Darbyshire et al., 2019).

Our findings support many of Mammen et al.’s (2023) conclusions, for example that NQNs expect to work in an environment of mutual respect and support, but are frequently disappointed, and NQNs are subject to a range of undermining, disempowering interactions from more experienced colleagues and managers. These experiences are associated with ‘resignation ideation’ (Urban et al., 2023), which was articulated directly and indirectly by our participants, some of whom planned to leave the profession because of their emotional distress, poor working conditions and low job satisfaction.

A spiral may arise where high turnover is a factor in decisions to leave, creating further turnover and worsening staffing levels (Gedik et al, 2023; Lee and Gagne, 2022). Our findings suggest that decreased confidence for NQNs was associated with poor delegation skills; similar findings were reported by Magnusson et al. (2017) who carried out an analysis of delegation behaviour styles among NQNs leading to increased workload and stress levels. Lack of confidence also meant that NQNs struggled to make decisions, manage clinical situations and were more likely to adopt a cautious approach when challenging unsafe practice (Mansour & Mattukoyya, 2019). Consequences are far-reaching: there are increased risks of morbidity and mortality for patients (Cline et al. 2022), and the NQNs in this study feared professional and emotional repercussions should omission of an intervention, caused by workload and time pressure, led to patient deterioration or even death. NQNs need supportive, safe relationships to learn, so they can build on their skills and develop confidence; in the absence of this, confidence is undermined, making learning more difficult and delaying development of competence and confidence (Daws et al. 2020). It is widely recognised in the pre-pandemic literature that decreased confidence, feelings of stress, and isolation are consequences of heavy workloads (Craig and Machin, 2020) compounded by inadequate support and low expectations from colleagues (Collard et al. 2020; Fawkes and Morre, 2019). It appears that pandemic-related factors may have exacerbated the high workload/low support dynamic experienced by this cohort, and so postponed or prevented the growth in confidence usually experienced by NQNs over time.

The NQNs in this study recounted their commitment to learning and becoming competent practitioners, to being able to deliver care and gain respect from colleagues they entered into the preceptorship period with the understanding of a psychological contract. A ‘psychological contract’ refers to unwritten mutual expectations and responsibilities in the workplace (Jacobs et al., 2020). When support to meet their professional and emotional needs was not forthcoming, their trust in senior colleagues was broken. This is congruent with a breach of the psychological contract. In common with participants in other studies (e.g. Jacobs et al., 2020), some NQNs considered leaving their profession. However, participants who felt safe in their roles (within supportive teams) expressed no intention to leave.

Nurse resilience can be strengthened by organisational support, for example by longer preceptorship time (Lee and Gagne, 2022). Labrague and de los Santos (2020) recommended a multi-faceted training programme for NQNs to improve work–life balance and communication. Cline et al. (2022) recommended a form of reflection in which NQNs were encouraged to acknowledge ‘three good things’ in a day, as a means of reducing stress. Sampson et al. (2020) evaluated a Cognitive Behavioural Therapy intervention which improved NQNs’ lifestyle behaviours and health. Although it is interesting that some interventions have an impact on enabling NQNs to cope, it is perhaps more revealing that the burden of dealing with harmful work pressures and cultural norms are commonly assumed to fall on individuals in a vulnerable position within organisations. The findings show the negative and costly impact, for NQNs themselves, and their employing organisations, of not being supported by an experienced clinician, with whom NQNs can feel emotionally safe, but we must recognise that the pandemic exacerbated stressful conditions experienced by all staff working in the NHS (Jones et al., 2024). Individual focus on professional accountability of established staff must be balanced with an organisational focus, which moves away from blame and supports a listening and learning culture (Jones et al., 2024). Dewey et al (2020) suggested that managing the workplace culture positively could facilitate best outcomes for both staff and patients.

NHS England (2023) recommended a series of interventions to promote ‘settling in’: continuing supernumerary status in the early weeks of practice; night shifts not being allocated in the first month; access to peer, mentors and buddy support, alongside more formal one-to-ones; daily team huddles which can be used for feedback, discussion and support; access to health and well-being resources and careful allocation in appropriate areas which match skills and competencies.

The participants in this study will become the preceptors and colleagues of the next cohorts and generations, many of whom will have experienced educational consequences of the pandemic during their teenage years. They conveyed a thoughtful committed approach to moving forwards, they had reflected on what they had missed out on and what needed to change, particularly in relation to nursing culture, for future NQNs. The COVID generations may require additional guidance as they navigate the reality of becoming the support they would have liked to have experienced themselves.

Previous studies have suggested that desirable preceptor characteristics include effective interpersonal, communication and teaching skills and the ability to provide useful feedback (Hardie et al., 2022), whereas Dewey et al. (2020) recommended that nurses be taught and supported in openly articulating emotional vulnerability. Alshawush et al. (2021) and Kukkonen et al (2025) concluded that support from preceptors and education on coping skills were beneficial in transition programmes for NQNs. They also noted that preceptors needed effective training in the role themselves.

Support and feeling valued, with the opportunity to increase knowledge and skills impacted positively on NQNs’ self-confidence and assertiveness, creating a sense of empowerment (Sahay et al., 2015). Feeling empowered was associated with independent decision-making and professional negotiation skills. Similar findings were reported in a qualitative study by Mansour and Mattukoyya (2018), which explored NQNs experiences of challenging unsafe practices. A meta-synthesis demonstrated that an environment which supports learning improves confidence, expands networking opportunities and maintains high standards of nursing care (Mlambo et al., 2021).

Confidence was also associated with exposure to positive role models, Craig and Machin (2020) described how NQN’s sense of self-efficacy had grown from observing to emulating more experienced nurses. Mansour and Mattukoyya (2019) argued that senior nurses, who act as inspiring role models and create a supportive and safe working culture, can promote confidence and provide valuable opportunities which will ensure NQNs reach their professional potential. Duchscher and Windey (2018) suggested that successful transition cannot necessarily be credited to support alone, as NQNs confidence improved naturally with time and exposure. By the end of the transition period, most NQNs appeared to have learnt from some of their challenging experiences, improved their competence and managed to overcome feelings of low confidence.

### Learning and recommendations

This study adds to existing evidence around the experiences of NQNs, with a focus on the COVID generation. Although it may be unlikely that those circumstances will arise again, the findings suggest interventions that could improve experiences for future cohorts, including those whose schooling was impacted by the pandemic.

Nurse education should provide opportunities for final-year nursing students to practise delegation skills, assertiveness and interpersonal communication for boundary setting; these should be reinforced in final-year placements (Jestico 2025).
Service leaders and managers should enforce existing zero tolerance policies related to workplace bullying, with colleagues encouraged to see NQNs as assets to be retained.
A supportive workplace culture enables NQNs to thrive, so colleagues at all levels should be supported to contribute to culture change.
Further research to explore factors that enable positive culture change and how this can be embedded should be prioritised.

## Strengths and limitations

Triangulation (e.g. survey data to capture statistical comparisons) and use of additional data sources (such as the perspectives of experienced staff) would have strengthened the study but was not possible due to time constraints. Potential bias was possibly introduced through the sampling strategy, as NQNs may have been motivated to participate due to more extreme experiences or stronger feelings. This has been valuable learning, nevertheless.

Three group interviews were conducted in the first instance, followed by individual interviews (Table 2). The interviewer’s senior role in an NHS organisation, and concomitant perception related to her current responsibilities, plus, for all the researchers’, remembered experiences of both transition and preceptorship, may have impacted on interpretation of findings. To mitigate this, sense checking was frequently carried out within the wider research team to discuss what had been said, meant, and interpreted.

The interview schedule was not piloted before data collection; this is acknowledged as a further limitation. To maintain consistency in data collection, iterative modifications were not made to the interview schedule. The interview schedule was closely aligned with the aims and objectives of the study, and guided by findings from the literature review conducted to inform it (Smythe and Carter, 2022).

Member checking was not carried out, this may have strengthened the credibility of the study, by ensuring that participants’ experiences were accurately interpreted (Ahmed, 2024). Not member checking transcriptions was not considered to be problematic as the recordings and transcriptions were checked rigorously by the research team who immersed themselves in the data. Furthermore, similar conclusions from earlier studies support our insights.

## Conclusion

The findings suggested that NQNs should be provided with opportunities for professional development and interventions should be implemented which recognise NQN status and include strategies to promote NQN resilience. There should be access to appropriate and supported mentors, buddies, peer support and a well-planned and structured approach to formal preceptorship. Effective support and training should be provided for nurses in preceptorship roles, particularly for those who may not have had good preceptorship modelled to them because of COVID-19 pressures. Further research is required to explore the impact of approaches which recognise NQN status.

**Key points for policy, practice and/or research**

Established staff require guidance on promoting a culture where new colleagues feel safe and develop confidence. This should include how to recognise behaviours that may be experienced as undermining, how to address them in self and others, and how to anticipate the anxieties of new colleagues and step up with guidance as required.
NQNs require protected opportunities to access support which is provided, whereas established colleagues need protected time to provide this support.
Future cohorts will have experienced disrupted secondary education due to COVID and may need more support and guidance than previous cohorts. This may include development of ‘soft skills’, such as collaborative working, conveying mutual respect in the workplace, prioritisation, and time management.
Ongoing training (e.g. in influencing and leading, creating positive team dynamics, managing self and others) should be provided for NQNs as they progress in their career, particularly for those who may not have had good preceptorship modelled to them.
The findings have potential to inform strategies aimed at staff well-being and retention.
